# Development and commissioning of a broadband online X-ray spectrometer for the SXFEL Facility

**DOI:** 10.1107/S1600577524005812

**Published:** 2024-07-29

**Authors:** Zhicheng Yang, Ximing Zhang, Heping Geng, Jiahua Chen, Chao Feng, Bo Liu, Bin Li

**Affiliations:** a Shanghai Institute of Applied Physics Chinese Academy of Sciences Shanghai201800 People’s Republic of China; b University of Chinese Academy of Sciences Beijing100049 People’s Republic of China; c Shanghai Synchrotron Radiation Facility, Shanghai Advanced Research Institute Chinese Academy of Sciences Shanghai201204 People’s Republic of China; d School of Physical Science and Technology ShanghaiTech University Shanghai201210 People’s Republic of China; University of Essex, United Kingdom

**Keywords:** free-electron lasers, online grating spectrometer, SUD beamline, e-TOF spectrometer, energy resolution, pulse length

## Abstract

A broadband online X-ray spectrometer has been designed and commissioned at the SUD beamline of the Shanghai Soft X-ray Free-Electron Laser Facility, which can deliver both SASE and seeded FEL pulses to user experiments, spanning the photon energy range of 50–620 eV. The resolving powers of the spectrometer calibrated via online measurement at 92 eV and 249 eV are ∼20000 and ∼15000, respectively, and the absolute photon energy is characterized by an electron time-of-flight spectrometer. The high energy resolution provided by the spectrometer can differentiate the fine structure in the FEL spectrum, to determine its pulse length.

## Abstract 2

A high-energy-resolution X-ray spectrometer has been commissioned at the SXFEL Facility, delivering single-shot resolving power beyond 20000 to provide support for fine-tuning and optimization of the machine, during scientific research. The fine structure of the individual spiky peak in the SASE spectrum is clearly resolved to characterize the pulse length of FEL radiation.

## 1. Introduction

In general, the X-ray free-electron laser (XFEL) can be operated in two major modes (Emma *et al.*, 2010 ▸; Feldhaus, 2010 ▸; Huang & Lindau, 2012 ▸; Ishikawa *et al.*, 2012 ▸), one is called self-amplified spontaneous emission (SASE) (Saldin *et al.*, 1998 ▸), and the other is seeding mode, including the schemes of self-seeding (Geloni *et al.*, 2011 ▸; Amann *et al.*, 2012 ▸) or seeding by external radiation sources (Allaria *et al.*, 2012 ▸; Penco *et al.*, 2013 ▸; Ratner *et al.*, 2015 ▸). Each XFEL pulse in either of these modes exhibits distinct characteristics, displaying various features shot-by-shot. Therefore, the ability to precisely measure and characterize the XFEL pulse in single-shot is in great demand, to provide feedback for commissioning and optimization of the XFEL machine, and to offer reliable light source parameters to scientific users, aiding normalization of experimental data and correction of the jitter in pulse energy, spectrum, temporal profile *etc*.

An X-ray spectrometer has two crucial parameters: one is the measurement photon energy range, and the other is the ultimate energy resolution. In particular, a SASE pulse contains multiple individual radiation peaks, associated with the features of a partially coherent light source. Its radiation spectrum contains multiple individual peaks as well, and according to the property of a partially coherent light source, the energy bandwidth of a typical individual peak (or single spike) in the spectrum is correlated to the XFEL pulse length. Thus, the high-resolution spectral measurements could also be used to calibrate the pulse duration of an XFEL light source in single-shot acquisition, which is a much easier technique for XFEL pulse length measurement compared with complicated XFEL timing diagnostics. The entire energy bandwidth of a SASE pulse, including all the individual single spikes, corresponds to the coherence time of the XFEL pulse. In the case of seeding mode, the radiation pulse is near full coherence and can be assumed to possess a Gaussian-like profile. XFELs have already been applied and played important roles in various research fields, such as condensed matter physics (Mankowsky *et al.*, 2014 ▸), advanced materials and surface science (Wernet *et al.*, 2015 ▸), atomic/molecular physics and chemistry (Takaba *et al.*, 2023 ▸), and molecular biology (Suga *et al.*, 2017 ▸). One of the most important diagnostic and measurement tools for these frontier research studies is high-resolution X-ray spectroscopy.

## 2. The SXFEL Facility

**Figure 1:**
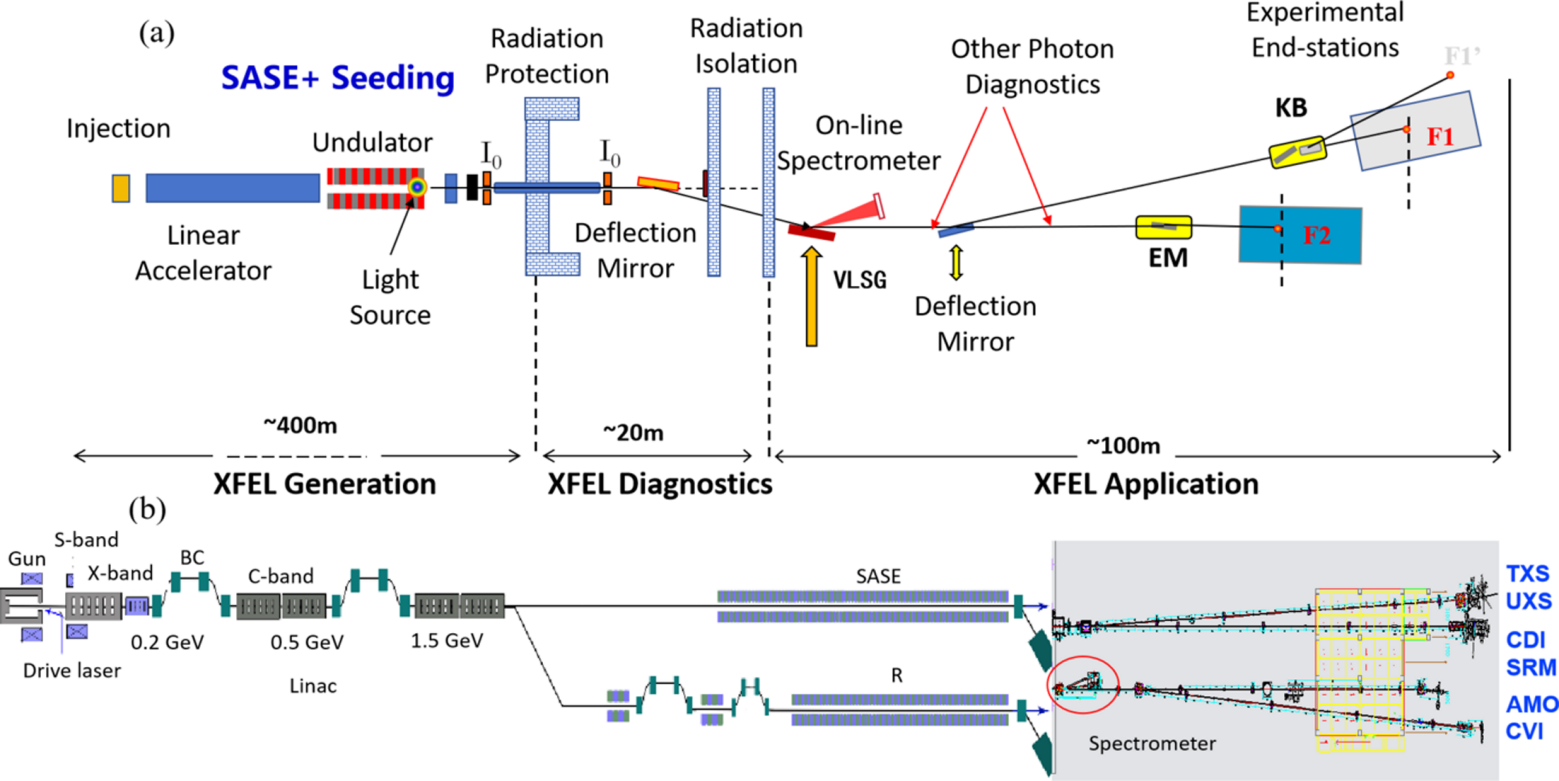
The development and commissioning of a broadband online X-ray energy spectrometer for the SXFEL Facility. (*a*) The overall layout and area divisions of the SUD beamline, consisting of three main parts: XFEL generation, XFEL diagnostics and XFEL application. (*b*) The engineering drawing shows the overall infrastructure of the SXFEL Facility, including accelerator division, SBP beamline, SUD beamline and six experimental end-stations.

The Shanghai Soft X-ray Free-Electron Laser Facility (SXFEL) (Zhao *et al.*, 2017 ▸) is one of the few operating soft XFEL facilities around the world, covering the entire water window (2.2–4.4 nm). The facility spans a total length of 532 m, and is equipped with two undulators followed by two separated X-ray photon beamlines and six experimental end-stations. As illustrated in Fig. 1 ▸, the SBP beamline delivers SASE pulses, housing ten in-vacuum undulator segments, where the undulator has a periodic magnet pole length of 16 mm and a total length of 4 m (IVU16), capable of generating SASE pulses with a minimum radiation wavelength of 2 nm. The seeded undulator division (SUD) beamline is able to operate in both SASE and seeding schemes, containing two modulated undulator segments, two dispersion units, six permanent magnet planar undulators (PMPU) with a pole period length of 30 mm and a length of 3 m for each undulator segment (U30), and ten PMPU with a pole period length of 23.5 mm and a length of 3 m for each segment (U235). The SUD beamline can generate fully coherent free-electron laser (FEL) pulses down to a shortest wavelength of 3 nm. The main parameters of the SXFEL Facility are summarized in Table 1 ▸.

The overall layout and area division of the SUD beamline for the SXFEL is shown in Fig. 1 ▸(*a*); it consists of three main components: XFEL generation, XFEL diagnostics and XFEL applications. The engineering diagram displaying the overall infrastructure of the SXFEL Facility, including the accelerator division, SBP beamline, SUD beamline and experimental stations, is shown in Fig. 1 ▸(*b*). The SXFEL Facility is designed and optimized to meet the fundamental requirements of scientific programs, including time-resolved X-ray scattering (TXS), ultrafast X-ray spectroscopy for chemistry (UXS), coherent diffraction imaging (CDI), live-cell fluorescent super-resolution microscope (SRM), atomic, molecular and optical dynamic imaging (AMO), composite velocity-map imaging spectrometer (CVI) *etc*.

This paper focuses on the design, development, commissioning and operation of online measurement of a broadband X-ray energy spectrometer at the SUD beamline of the SXFEL Facility, implementing a pair of variable line-spacing (VLS) gratings, indicated by a red circle in Fig. 1 ▸(*b*). The photoelectron spectroscopy generated by the same FEL pulses is measured by an electron time-of-flight spectrometer (e-TOF) installed on the upper stream of the SUD beamline.

## 3. Design of the online spectrometer

The soft X-ray spectral measurements typically employ either a grating-based technique (Harada & Kita, 1980 ▸; Kita *et al.*, 1983 ▸; Brenner *et al.*, 2011 ▸; Ragozin *et al.*, 2021 ▸; Zhang *et al.*, 2020 ▸; Li & Li, 2018 ▸, 2019 ▸) or a multilayer coating structure (Wang & Li, 2021 ▸; Voronov *et al.*, 2011 ▸, 2015 ▸; Louis *et al.*, 2011 ▸; Rife *et al.*, 1989 ▸). Here, the online spectrometer is installed on the SUD beamline of the SXFEL Facility, implementing a pair of VLS gratings to measure the XFEL radiation spectrum during the machine commissioning and operation. An e-TOF spectrometer is situated in the sub-branch of the SUD beamline, located at the upper stream, which is used to calibrate the central photon energy of the FEL radiation; and a 16 e-TOF detector array is integrated to measure the polarization of X-ray photons based on the angle-resolved photoelectron emission intensity and spectral distribution (Deng *et al.*, 2022 ▸; Braune *et al.*, 2016 ▸).

The VLS grating of the online X-ray spectrometer adopts an embedded ‘grating-mirror’ design, where the surface area mainly serves as an effective X-ray reflection mirror, while only a small central portion of the mirror surface is grooved as a grating region, to diagnose the first-order diffractive light. Simultaneously, the reflected component either from the flat mirror or from the grooved area (the zero-order beam) is redirected to the optical elements located downstream of the SUD beamline, maintaining the optical quality and wavefront of the X-ray beam.

The technical specifications for both gratings VLSG1 and VLSG2 used in the online spectrometer for the SUD beamline are listed in Table 2 ▸. The parameters are home designed and the gratings were manufactured by the Jtec Corporation. Grating VLSG1 is optimized for the energy range of 50–250 eV to deliver a resolving power of >20000, while grating VLSG2 is optimized for 200–620 eV to deliver a resolving power of >15000. The spectrometer spans the entire water window, particularly achieving meridional flat field for each measured photon energy range. During the measurement, the positions of the gratings are fixed, with a constant grazing-incidence angle of 2° for the whole energy range. By fine-tuning and optimizing the coordinate and tilt angle of the CCD detector, real-time single-shot X-ray spectral measurements at different photon energies (wavelengths) can be achieved, ensuring high energy resolution over the entire spectral range. The two gratings are installed on a unique mirror mount, and their front surfaces share a common rotation axis, which facilitates the manipulation and fine-tuning of the gratings.

**Figure 2:**
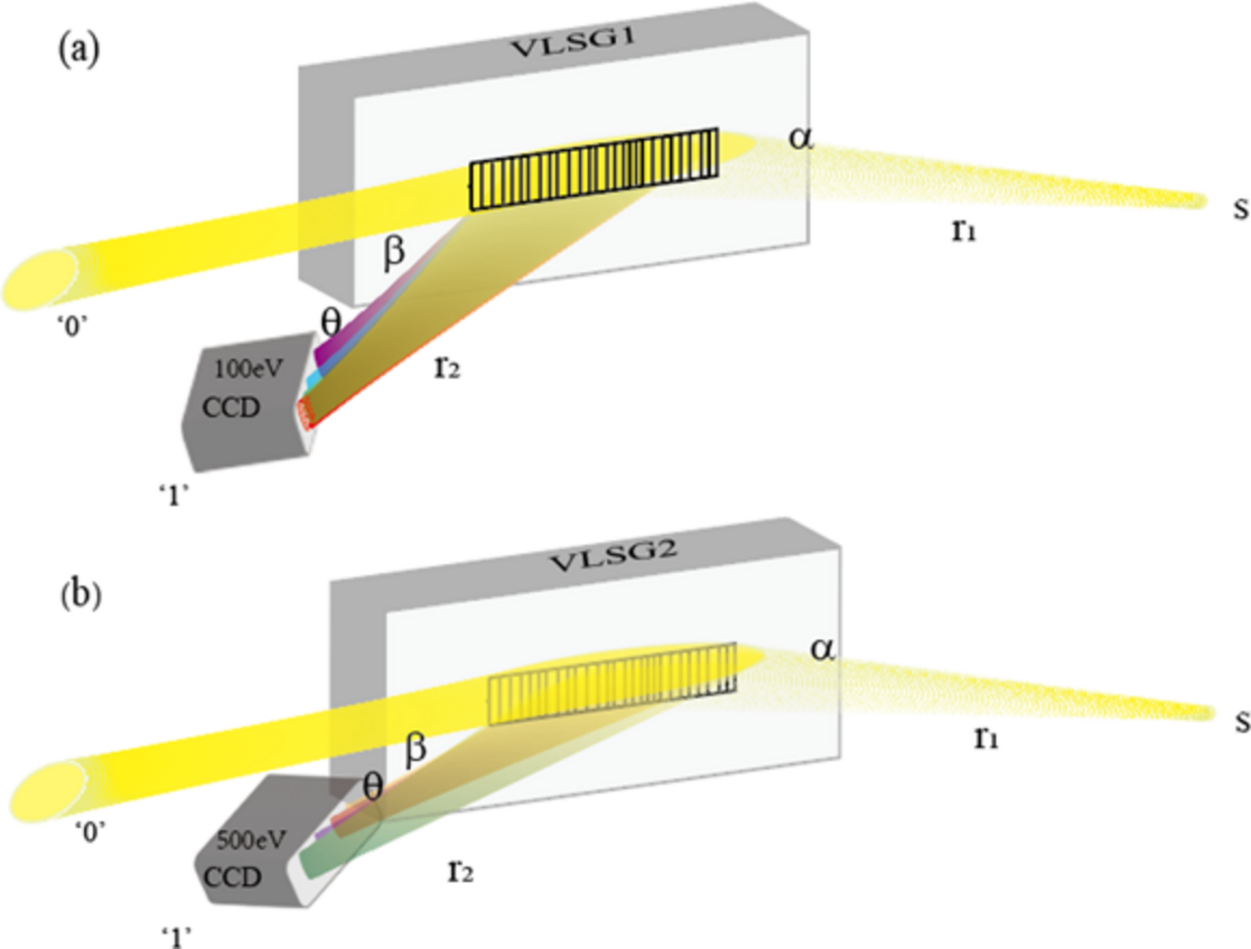
The schematic diagram shows the working principle of the SXFEL online spectrometer. (*a*) The grating VLSG1 operates at a photon energy of 100 eV, where the detector plane is set at an angle of 73.3° with respect to the first-order diffraction beam. (*b*) The grating VLSG2 operates at a photon energy of 500 eV, where the detector plane is at an angle of 24.6° with respect to the first-order beam.

**Figure 3:**
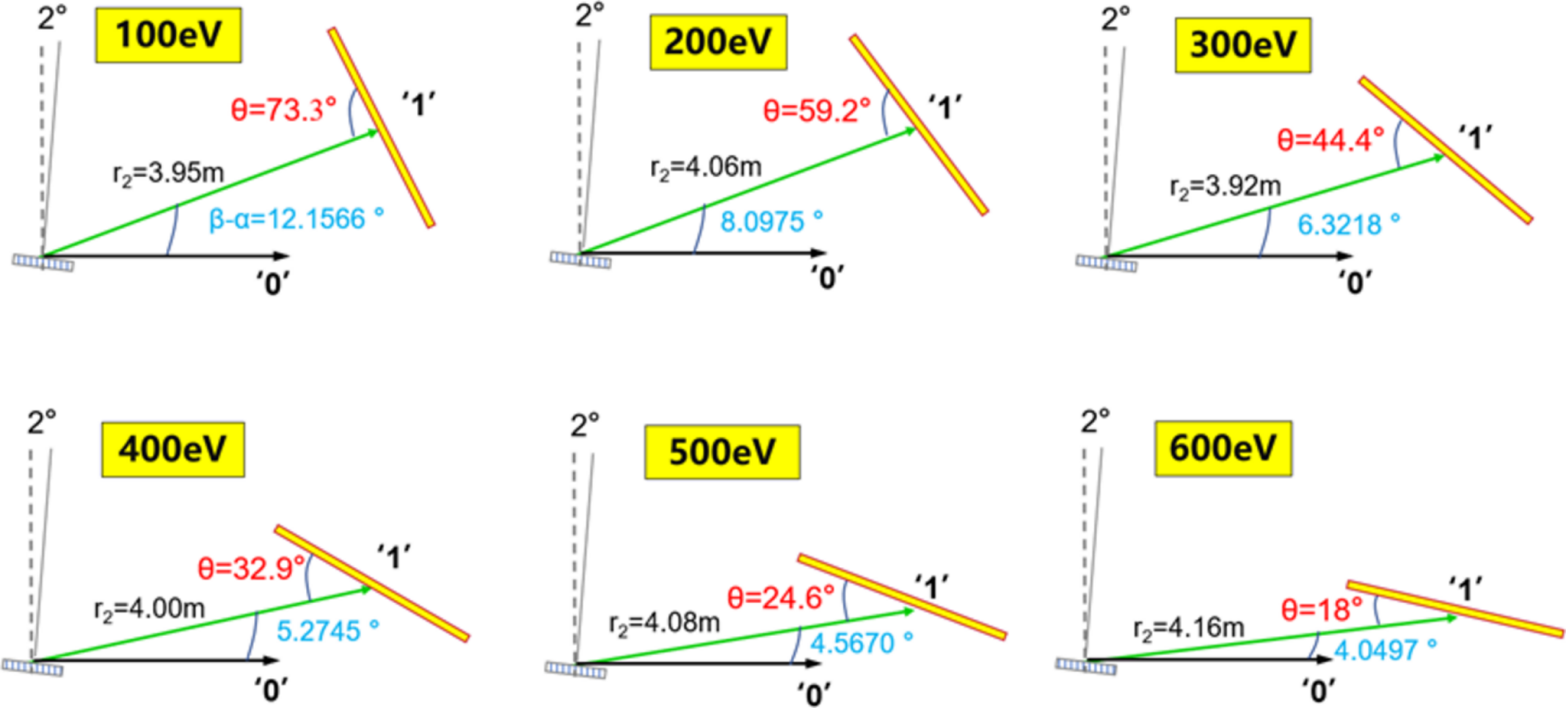
The optimal geometry for the detector of the SXFEL online spectrometer is illustrated, where the desired focal length and orientation angle θ (red) of the detector plane with respect to the first-order beam at various energies are shown. The dashed line in each sub-plot is perpendicular to the propagation direction of the 0th-order beam, while the thin solid line represents the surface normal of the grating.

Fig. 2 ▸ is a schematic diagram showing how the SXFEL online diagnostic spectrometer works, utilizing gratings VLSG1 or VLSG2. The spectrometer works as an online diagnostic; thus, the grating is immobile during the measurement, and the grazing-incidence angle is fixed to 2° for the entire photon energy range. The source point of the spectrometer is assumed to be at the end of the last undulator segment, *i.e.* at a distance of *r*_1_ = 59 m. The image distance is the spatial separation in-between the detector and the center of the grating, which is *r*_2_ = 3.85–4.25 m for different photon energies. In Fig. 2 ▸(*a*), the schematic geometry for grating VLSG1 working at 100 eV is illustrated, with the ‘0’ order accounting for ∼90% of the total beam intensity, delivered downstream for user experiments; meanwhile, the ‘1’ order with 7–8% of the total beam intensity is delivered to a CCD detector for online spectral measurement. In order to achieve the best spectral resolution at the current photon energy, the detector is set to coincide with the meridional focal curve in space, by optimizing both the focal length (*i.e.* image distance) and the reception angle of the detector with respect to the diffraction beam, which is 73.3° at 100 eV. Fig. 2 ▸(*b*) shows a schematic for the VLSG2 operating at 500 eV; the ultimate spectral resolution is achieved similarly by tuning the image distance and the orientation of the detector plane in space, where the intersection angle in-between the ‘1’ order diffraction beam and the CCD detector plane is only 24.6°. It is worth pointing out that, in order to achieve the best performance of the spectrometer, the diffraction beam should be set to tilt more to grazing incidence to the detector plane for higher photon energies (refer to Fig. 3 ▸).

The VLS grating focusing equation is given by
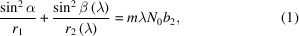
where α is the fixed grazing-incidence angle at 2°, β is the first-order grazing diffraction angle (complementary to the primary diffraction angle, wavelength dependence), *m* is the diffraction order (for the first-order diffraction, *m* = 1), and *N*_0_ is the groove density of the grating at the center, which is 2400 lines mm^−1^ in our case.

Adopting the above notation, the grating equation is
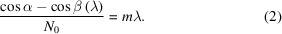
Then, the image distance (for various wavelengths) can be derived as
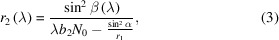
where the first-order grazing diffraction angle is β(λ) = arccos (cos α − *N*_0_ λ), according to equation (2), *b*_2_ is the linear coefficient (or the second order) of the VLS law for the grating (refer to Table 2 ▸).

For any given wavelength, the ideal intersection angle θ in-between its first-order diffraction beam and the detector plane can be calculated using the following formula (Osborn & Callcott, 1995 ▸):
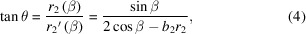
where 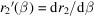 is the first-order gradient of the outgoing diffraction beam with respect to the current diffraction angle (Zhang *et al.*, 2020 ▸).

Utilizing equations (3) and (4), the optimal detector position, *i.e.* image distance, and the ideal intersection angle in-between the detector plane and the central diffraction beam for different photon energies in the measurement range can be determined; the specifications for 100–600 eV are shown in Fig. 3 ▸. In each sub-plot, the green line represents the diffraction arm at the first order, the focal length (image distance) for each photon energy is given, which is more or less than 4 m, θ is the intersection angle between the detector plane and the diffraction arm (marked in red). The dashed line is perpendicular to the zero-order beam propagation direction, while the thin solid line represents the surface normal of the grating plane. The position of the CCD detector can be optimized by adjusting focal length and the angle θ, to deliver the best energy resolution at different energies.

**Figure 4:**
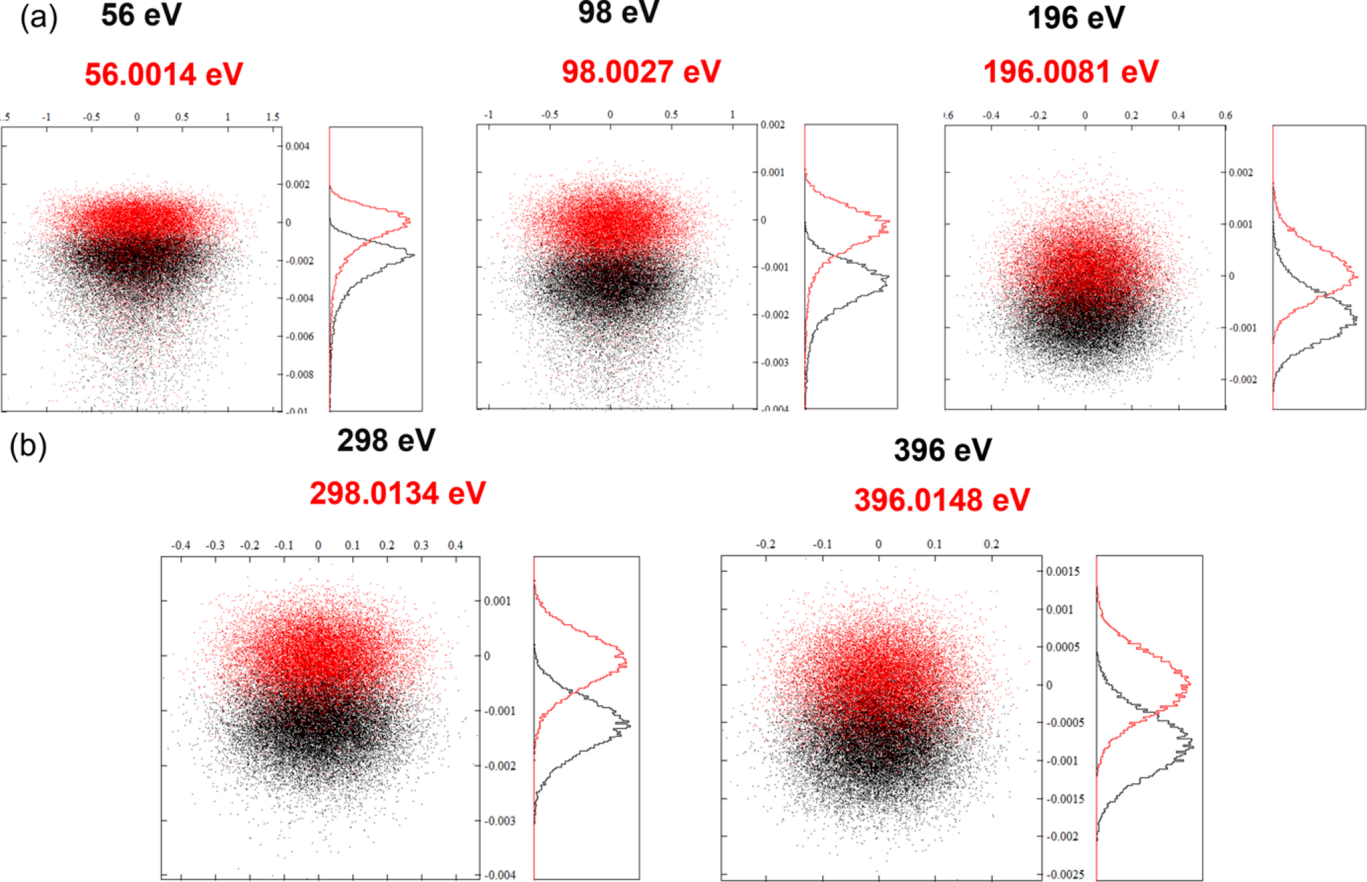
Ray-tracing by the *XOP Shadow* program is used to evaluate the theoretical resolving power at different energies. The tracing results of VLSG1 (*a*) and VLSG2 (*b*) at typical photon energies are shown.

Ray-tracing results at different energies obtained using the *XOP Shadow* program (Sanchez del Rio *et al.*, 2011 ▸) are shown in Fig. 4 ▸. The surface slope error of the grating is set to 0.5 µrad in the meridian coordinate and 2 µrad in the sagittal coordinate, which are a bit larger than the realistic fabrication errors provided by Jtec (Table 2 ▸), leaving some margin for the practical procedure from simulation. Fig. 4 ▸(*a*) shows ray-tracing results for grating VLSG1 at a few representative energies, while Fig. 4 ▸(*b*) shows results for grating VLSG2. The black and red patterns in each plot are associated, respectively, with the investigated photon energies and an energy slightly deviated away from the center, which are distinguishable. The best resolving powers by grating VLSG1 (*E*/Δ*E*) are 24000–36000 at the several selected photon energies, while the resolving powers of grating VLSG2 are 20000–28000. The simulated and preliminarily calibrated source size and beam divergence for photon energy spanning the range 50 eV to beyond 600 eV are considered in the above ray-tracing procedure, *e.g.* at 98 eV, the source size is ∼380 µm (r.m.s.) and the divergence angle is ∼44 µrad (r.m.s.); at 298 eV, the source size is ∼270 µm (r.m.s.) and the divergence angle is ∼18 µrad (r.m.s.).

The SUD beamline is also equipped with an X-ray coherence measurement device, allowing for measurements of both the wavefront coherence and longitudinal coherence of X-ray pulses. However, in order to calibrate the longitudinal (temporal) coherence or the pulse length, this technique relies on delay scanning and cannot achieve single-shot resolution. Therefore, we have developed the online THz streaking (Helml *et al.*, 2017 ▸; Wieland *et al.*, 2021 ▸) apparatus on the beamline, which can provide the pulse length of an XFEL in single-shot mode. However, we have not yet achieved this; currently an e-TOF spectrometer has been installed and commissioned in the THz streaking device for future applications. The central photon energy of the FEL radiation and its shot-by-shot jitter can be measured and monitored, which provides an indispensable reference for the energy calibration of the online spectrometer.

## 4. Experiment and discussion

**Figure 5:**
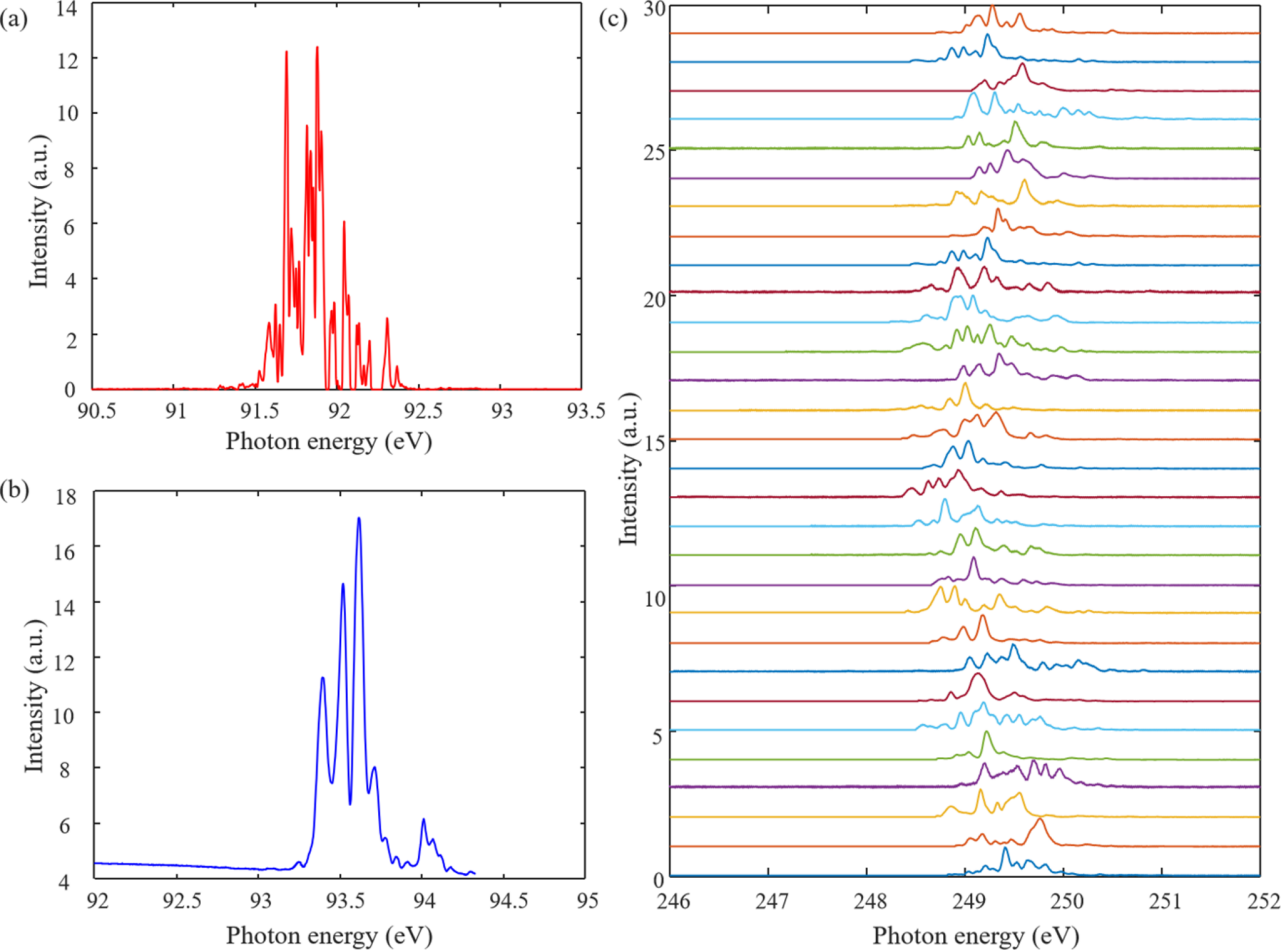
Single-shot spectral measurement of FEL pulses in SASE mode. (*a*) Typical SASE pulse spectral measurement at ∼92 eV for a longer pulse width. (*b*) The SASE pulse spectral measurement at ∼92 eV for a shorter pulse width. (*c*) The spectral measurement for 30 sequential single-shot FEL pulses in SASE mode, at ∼249 eV.

The online X-ray spectrometer has been installed and commissioned on the SUD beamline of the SXFEL Facility. The measured spectra at several representative cases, implementing grating VLSG1 or VLSG2, are shown in Fig. 5 ▸. In Fig. 5 ▸(*a*), a single-shot SASE spectrum at ∼92 eV (λ ∼ 13.5 nm) is shown, consisting of multiple narrow individual spikes. Among them, the finest distinguishable feature only occupies less than 2 pixels on the CCD detector; the projection angle of this fine feature with respect to the center of the grating can be estimated by Δβ ∼ 2 × 13 µm × [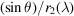] = 6.36 µrad. The corresponding wavelength range spanning the above projection angle is Δλ ∼ *d*_0_ × 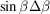 = 6.7561 × 10^−13^ m. Thus, the energy resolution at ∼92 eV can be calculated, *E*/Δ*E* = λ/Δλ ∼ 19982. This outlines the simple procedure to evaluate the energy (spectral) resolution of the X-ray spectrometer. At ∼92 eV, the energy resolution approaches ∼20000, which is a bit lower compared with the simulation value of >30000, according to the ray-tracing program (in Fig. 4 ▸). Obviously, one of the most important things when calibrating the energy resolution is to find out and identify the finest structure from piles of single-shot SASE spectra. The sharpest distinguishable feature of 2 pixels probably represents a conservative assessment. If the finest differentiable feature is taken to be contained within 1 pixel, the corresponding resolving power is ∼40000. Now, when we consider the other factors limiting the resolution of the spectrometer, *e.g.* the optical fabrication errors and the optical aberration terms of the system, the ultimate resolution would drop a bit, being comparable with the simulation result displayed in Fig. 4 ▸.

In Fig. 5 ▸(*a*), the typical bandwidth full width at half-maximum (FWHM) for a significant spiky peak in the SASE spectrum is also characterized, which is Δ*E* (FWHM) ∼ 0.032 eV. Under a Gaussian pulse assumption, the transform-limit pulse length (Reiche *et al.*, 2008 ▸; McNeil *et al.*, 2013 ▸; Nicolas & Cocco, 2022 ▸) is about 133.3 fs (r.m.s.), *i.e.* ∼314 fs (FWHM). On another day, the online spectrometer was operated to measure the spectrum around ∼92 eV when the electron bunch length is more compressed, and the result is presented in Fig. 5 ▸(*b*) (notably, the central photon energy shifted towards higher energy to about 93.5 eV). It is apparent that the spectrum includes fewer spikes, much less than the number in Fig. 5 ▸(*a*); compared with the feature in Fig. 5 ▸(*a*), the bandwidth of a single spike appears much broader, which is about Δ*E* (FWHM) ∼ 0.064 eV, associated with the transform-limit Gaussian pulse length, ∼67.4 fs (r.m.s.), *i.e.* ∼159 fs (FWHM). The radiation pulse lengths for these two cases are compatible with the bunch parameters provided by the accelerator division of the SXFEL Facility.

Furthermore, the spectrometer implements VLSG2 to measure the energy spectrum around ∼5 nm (∼249 eV) in SASE mode. The acquisition of 30 consequential single-shot spectra is displayed in Fig. 5 ▸(*c*). Using a similar procedure discussed in the case of ∼92 eV, the energy resolution at ∼249 eV can be derived, which is about *E*/Δ*E* = λ/Δλ ≃ 15052; in the meantime, the typical bandwidth for the dominant spike in the spectrum is identified as well, corresponding to a transform-limit pulse length of ∼102 fs (FWHM), with r.m.s. uncertainty of 24 fs. According to the result in Fig. 5 ▸(*c*), we can also evaluate the photon energy jitter at ∼5 nm, where the mean photon energy is 249.26 eV and the photon energy jitter is 0.23 eV (r.m.s.). Thus, the ratio of the standard deviation to the average value of the 30 pulses is about 9.2 × 10^−4^ (r.m.s.). However, since the FEL beam has pointing instability, this would contribute to the energy jitter as well. Given a 10% beam position and pointing instability, the estimated beam pointing jitter should be <3 µrad (r.m.s.) at 5 nm, causing an additional energy jitter of 4.0 × 10^−5^ (r.m.s.), which is negligible compared with the overall spectral jitter calibrated during the operation of the VLSG2 spectrometer for this short-term run. We conducted quite a few experimental runs, and eventually confirmed that the single-shot photon energy jitter for this SASE mode at 5 nm should be <1.0 × 10^−3^ (r.m.s.), which accounts for ∼0.1% (r.m.s.) with respect to the average photon energy, indicating the shot-by-shot radiation spectral jitter is small.

The current spectral measurements were carried out on the SXFEL Facility running at a repetition rate <10 Hz; in the near future, the facility will be upgraded to deliver a higher repetition rate, *e.g.* 50 Hz. By then, we will have to improve the readout frame rate of the CCD detector to keep pace with the FEL repetition rate to avoid multi-shot data overlapping, which would blur the fine features in the spectrum, severely limiting our ability to achieve the ultimate spectral resolution.

**Figure 6:**
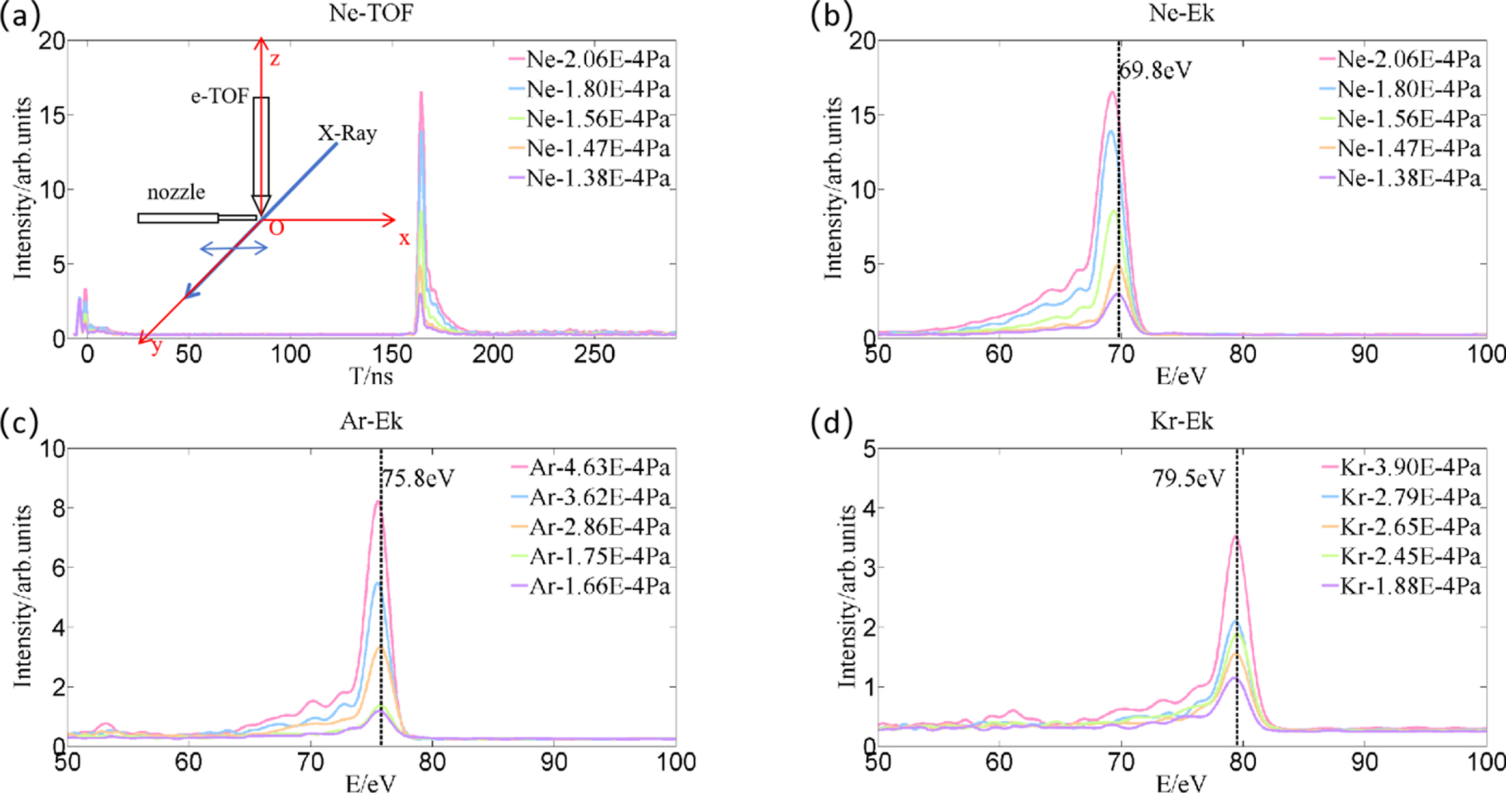
The photoelectron energy spectrum measurements using different noble gases via the e-TOF apparatus. The inset schematic diagram shows the coordinates for the X-ray beam path, gas injection nozzle and e-TOF device in the experimental setup. (*a*) The cumulative photoelectron time-of-flight spectra for 1000 shots in neon gas at different pressures. (*b*), (*c*) and (*d*) are the corresponding cumulative photoelectron kinetic energy spectra for 1000 shots in neon, argon and krypton at different pressures.

For the above cases, the photon energy is calibrated through measuring and identifying the position of each pixel at the CCD detector, with respect to the center of the grating located in the online spectrometer. Thus, the accuracy of energy calibration is purely reliant on the precision of spatial measurement in distance and angle. After double-checking the parameters of the machine, *e.g.* the kinetic energy of the electron bunches, the magnetic pole gap of the undulator segments during the operation, we find out this method works quite well, achieving values similar to those of the accelerator division. In order to ensure the reliability of photon energy calibration for the grating spectrometer, we also refer to an e-TOF spectrometer to measure the photoemission spectra in gases simultaneously. Fig. 6 ▸ shows the photoelectron energy spectra of several rare gases measured with the e-TOF spectrometer. Fig. 6 ▸(*a*) plots a 1000-shot accumulative photoelectron time-of-flight spectrum in neon gas with different pressures, excited by FEL pulses at ∼13.5 nm, possessing horizontal polarization. The inset schematic shows the working scheme of the e-TOF device; the spectra mainly consist of the photoexcited electrons from neon’s valence band, and the two small peaks located at the left end in the spectral range correspond to optical scattering signals – the first one from the left is attributed to the *Bremsstrahlung* radiation generated in the accelerator section at the upper stream of the facility, usually possessing tens to hundreds of MeV kinetic energy, while the second one is the FEL photon scattering signal from the gas jet in the interaction regime. The latter is created while the FEL beam passes close by the gas injection nozzle with an aperture of about 300 µm; obviously it is affected more by the gas pressure in the chamber, compared with the former. Therefore, the second optical signal is assigned as the time zero in the e-TOF spectrometer, and further used to correct the flight time to calibrate the kinetic energy for photoelectrons.

In Figs. 6 ▸(*b*)–6 ▸(*d*) the photoelectron energy spectra of neon, argon and krypton gases at various pressures are presented. Since our measurement is within the energy range of 10–500 eV, corresponding to the best performance energy range for the e-TOF, it is unnecessary to apply the retardation potential to improve the resolution. The photoelectron’s kinetic energy *E_k_* can be derived straightforwardly from the photoelectron’s flight time by equation (5), 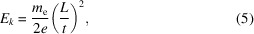
where *m*_e_ is the electron mass, *L* is the photoelectron drift length, *t* is the electron flight time, *e* is the electron’s charge.

For Fig. 6 ▸(*b*), it is known that the electron binding energy of the 2*p* orbital in the Ne atom is 22 eV, and the photoemission peak is located at the energy of 69.8 eV, so the FEL photon energy used in the measurement is about 91.8 eV. Upon removing and clearing neon from the chamber and gas injection pipes, the measurement is carried out again after refilling with argon gas and stabilizing pressure. As shown in Fig. 6 ▸(*c*), the electron binding energy of the 3*p* orbital in an Ar atom is 16 eV and the peak energy is 75.8 eV, so the FEL photon energy is calculated as 91.8 eV. Photoelectron spectral broadening is observed when the pressure is higher, due to a stronger scattering process during the ionization, which also leads to a slight energy shift. Thus, choosing the right or appropriate gas pressure for the photoemission experiment is critical. Similarly, on another day, we conducted the e-TOF measurement using Kr, and obtained the FEL photon energy as 79.5 + 14.1 ≃ 93.6 eV, shown in Fig. 6 ▸(*d*). Since the photoemission experiments by e-TOF were carried out with the grating-based X-ray spectrometer during the same beamtime slots but on two different days, we can see that the measurements by the two diagnostics are basically in agreement with each other, *i.e.* Figs. 6 ▸(*b*)–6(*c*) versus Fig. 5 ▸(*a*), and Fig. 6 ▸(*d*) versus Fig. 5 ▸(*b*). Thus, the e-TOF device can be used to calibrate the central photon energy of the FEL radiation. It is worth pointing out that, in the above photoemission measurements, only the electrons excited from *p* orbitals in the valence band were observed, not those from *s* orbitals, which is mainly due to the selection rule and electron distribution in the photon-ionization process. The *s* → *p* photoexcitation leads to the electrons in the final state being mainly ejected along the direction of the FEL polarization, which is perpendicular to the axis of the e-TOF device, so they have a rare chance of entering into the e-TOF and being captured by the multi-channel plate detector at the end of the flight tube; however, in the *p* → *s* photoexcitation, the electrons in the final state are distributed within the *s* cloud, possessing approximately spherical symmetry, *i.e.* the ionization orbital could extend in all directions, more or less homogeneously distributed in space, thus producing a much stronger and observable photoemission peak intensity in the spectrum. This result is consistent with previous theoretical calculations (Trzhaskovskaya *et al.*, 2001 ▸).

## 5. Conclusion

A broadband grating-based X-ray spectrometer has been developed and installed online to support the commissioning and operation of the SXFEL Facility. The designated photon energy range spans ∼50 eV to ∼620 eV, which is beyond the entire water window. The single-shot FEL spectral measurements were carried out at the two representative photon energies of ∼92 eV and ∼249 eV, demonstrating the energy resolutions of ∼20000 and ∼15000, respectively.

The photon energy of the spectrometer is calibrated through precise measurement and identification of the position of each pixel at the CCD detector, with respect to the center of the grating. An electron time-of-flight spectrometer is adopted to calibrate the central photon energy of the radiation, cross-checking with the parameters provided by the accelerator division of the facility. The measurement results of all these methods are consistent with each other, indicating the precision and reliability of the energy calibration for the spectrometer.

A spectrometer with sufficiently high energy resolution is a decent online diagnostic device to monitor the single-shot spectral jitter for FEL radiation at various operation modes and in different photon energies. More remarkably, it could be implemented to reflect FEL pulse length in single-shot mode, which is much easier to use compared with XFEL timing diagnostics.

In the near future, the THz streaking apparatus including the e-TOF device will be prepared to measure the FEL pulse length. Then, we will be able to obtain more diagnostic data in real time, and have a better understanding of the correlation between the spectral and temporal parameters of the FEL radiation.

## Figures and Tables

**Table 1:** Parameters of the SXFEL Facility

Electron energy	1.5 GeV
Bunch normalized emittance	<1.5 mm mrad
Slice energy spread	<0.02%
Bunch charge/length/peak current	500 pC/50–200 fs/>1000 A
Wavelength/pulse length of the seed laser	265 nm (tunable) /∼100 fs
Repetition rate	10–50 Hz
FEL radiation pulse energy	∼50 µJ
FEL wavelength	2–15 nm
FEL pulse length	100–200 fs
FEL peak power	>100 MW
Spectral width of seeded FEL pulse (FWHM)	0.01%–0.03%
FEL beam size at undulator exit (FWHM)	100–300 µm
FEL beam divergence (FWHM)	10–60 µrad
FEL average flux	5×10^11^–10^13^ photons s^−1^
FEL peak brilliance	7×10^28^–1.5×10^30^ photons/[s (mm^2^) (mrad^2^) (0.1%BW)]

**Table 2:** The technical specifications of gratings VLSG1 and VLSG2

	VLSG1	VLSG2
Energy range	50–250 eV	200–620 eV
Substrate profile	Plain	Plain
Effective optical dimensions	280 mm (L) × 30 mm (W)	280 mm (L) × 30 mm (W)
Substrate dimensions	300 mm (L) × 40 mm (W) × 50 mm (T)	300 mm (L) × 40 mm (W) × 50 mm (T)
Fabrication error of substrate dimensions	± 0.2 mm	± 0.2 mm
Slope error of the substrate	<0.2 µrad r.m.s. (tangential)/<0.5 µrad r.m.s. (sagittal)	<0.2 µrad r.m.s. (tangential)/<1.0 µrad r.m.s. (sagittal)
Groove area	80 mm (L) × 5 mm (W)	40 mm (L) × 5 mm (W)
Grating parameters	*N*(*x*) = *N*_0_(1+*b*_2_*x*+*b*_3_*x*^2^+*b*_4_*x*^3^+…) where *x* is tangential coordinate of the grating, with respect to grating center in units of mm. *N*_0_ = 2399.664 mm^−1^; *b*_2_ = 5.109 × 10^−4^ mm^−1^; *b*_3_ = −1.895 × 10^−7^ mm^−2^; *b*_4_ = 1.746 × 10^−10^ mm^−3^	*N*(*x*) = *N*_0_(1+*b*_2_*x*+*b*_3_*x*^2^+*b*_4_*x*^3^+…) where *x* is tangential coordinate of the grating, with respect to grating center in units of mm. *N*_0_ = 2399.337 mm^−1^; *b*_2_ = 5.426 × 10^−4^ mm^−1^; *b*_3_ = −2.093 × 10^−7^ mm^−2^; *b*_4_ = 0.746 × 10^−10^ mm^−3^
Groove profile	Constant groove depth: *h* = 14 nm ± 15%; groove width/spacing: *w*/*d* = 0.65 ± 15%	Constant groove depth: *h* = 14 nm ± 15%; groove width/spacing: *w*/*d* = 0.65 ± 10%
Coating	B_4_C 40 nm ± 10 nm with Cr binding layer	Ni 40 nm ± 10 nm with Cr binding layer
Roughness on grooves	0.3 nm r.m.s.	0.3 nm r.m.s.

## References

[bb1] Allaria, E., Appio, R., Badano, L., Barletta, W. A., Bassanese, S., Biedron, S. G., Borga, A., Busetto, E., Castronovo, D., Cinquegrana, P., Cleva, S., Cocco, D., Cornacchia, M., Craievich, P., Cudin, I., D’Auria, G., Dal Forno, M., Danailov, M. B., De Monte, R., De Ninno, G., Delgiusto, P., Demidovich, A., Di Mitri, S., Diviacco, B., Fabris, A., Fabris, R., Fawley, W., Ferianis, M., Ferrari, E., Ferry, S., Froehlich, L., Furlan, P., Gaio, G., Gelmetti, F., Giannessi, L., Giannini, M., Gobessi, R., Ivanov, R., Karantzoulis, E., Lonza, M., Lutman, A., Mahieu, B., Milloch, M., Milton, S. V., Musardo, M., Nikolov, I., Noe, S., Parmigiani, F., Penco, G., Petronio, M., Pivetta, L., Predonzani, M., Rossi, F., Rumiz, L., Salom, A., Scafuri, C., Serpico, C., Sigalotti, P., Spampinati, S., Spezzani, C., Svandrlik, M., Svetina, C., Tazzari, S., Trovo, M., Umer, R., Vascotto, A., Veronese, M., Visintini, R., Zaccaria, M., Zangrando, D. & Zangrando, M. (2012). *Nat. Photon.* **6**, 699–704.

[bb2] Amann, J., Berg, W., Blank, V., Decker, F.-J., Ding, Y., Emma, P., Feng, Y., Frisch, J., Fritz, D., Hastings, J., Huang, Z., Krzywinski, J., Lindberg, R., Loos, H., Lutman, A., Nuhn, H.-D., Ratner, D., Rzepiela, J., Shu, D., Shvyd’ko, Yu., Spampinati, S., Stoupin, S., Terentyev, S., Trakhtenberg, E., Walz, D., Welch, J., Wu, J., Zholents, A. & Zhu, D. (2012). *Nat. Photon.* **6**, 693–698.

[bb3] Braune, M., Brenner, G., Dziarzhytski, S., Juranić, P., Sorokin, A. & Tiedtke, K. (2016). *J. Synchrotron Rad.* **23**, 10–20. 10.1107/S1600577515022675 PMC4733935 26698040

[bb4] Brenner, G., Kapitzki, S., Kuhlmann, M., Ploenjes, E., Noll, T., Siewert, F., Treusch, R., Tiedtke, K., Reininger, R., Roper, M. D., Bowler, M. A., Quinn, F. M. & Feldhaus, J. (2011). *Nucl. Instrum. Methods Phys. Res. A*, **635**, S99–S103.

[bb5] Deng, B., Liu, Z., Yang, H., Zhang, Q., Deng, H. & Liu, B. (2022). *J. Instrum.* **17**, P05046.

[bb6] Emma, P., Akre, R., Arthur, J., Bionta, R., Bostedt, C., Bozek, J., Brachmann, A., Bucksbaum, P., Coffee, R., Decker, F.-J., Ding, Y., Dowell, D., Edstrom, S., Fisher, A., Frisch, J., Gilevich, S., Hastings, J., Hays, G., Hering, Ph., Huang, Z., Iverson, R., Loos, H., Messerschmidt, M., Miahnahri, A., Moeller, S., Nuhn, H.-D., Pile, G., Ratner, D., Rzepiela, J., Schultz, D., Smith, T., Stefan, P., Tompkins, H., Turner, J., Welch, J., White, W., Wu, J., Yocky, G. & Galayda, J. (2010). *Nat. Photon.* **4**, 641–647.

[bb7] Feldhaus, J. (2010). *J. Phys. B At. Mol. Opt. Phys.* **43**, 194002.

[bb8] Geloni, G., Kocharyan, V. & Saldin, E. (2011). *J. Mod. Opt.* **58**, 1391–1403.

[bb9] Harada, T. & Kita, T. (1980). *Appl. Opt.* **19**, 3987. 10.1364/AO.19.003987 20234726

[bb10] Helml, W., Grguraš, I., Juranić, P., Düsterer, S., Mazza, T., Maier, A., Hartmann, N., Ilchen, M., Hartmann, G., Patthey, L., Callegari, C., Costello, J., Meyer, M., Coffee, R., Cavalieri, A. & Kienberger, R. (2017). *Appl. Sci.* **7**, 915.

[bb11] Huang, Z. & Lindau, I. (2012). *Nat. Photon.* **6**, 505–506.

[bb12] Ishikawa, T., Aoyagi, H., Asaka, T., Asano, Y., Azumi, N., Bizen, T., Ego, H., Fukami, K., Fukui, T., Furukawa, Y., Goto, S., Hanaki, H., Hara, T., Hasegawa, T., Hatsui, T., Higashiya, A., Hirono, T., Hosoda, N., Ishii, M., Inagaki, T., Inubushi, Y., Itoga, T., Joti, Y., Kago, M., Kameshima, T., Kimura, H., Kirihara, Y., Kiyomichi, A., Kobayashi, T., Kondo, C., Kudo, T., Maesaka, H., Maréchal, X. M., Masuda, T., Matsubara, S., Matsumoto, T., Matsushita, T., Matsui, S., Nagasono, M., Nariyama, N., Ohashi, H., Ohata, T., Ohshima, T., Ono, S., Otake, Y., Saji, C., Sakurai, T., Sato, T., Sawada, K., Seike, T., Shirasawa, K., Sugimoto, T., Suzuki, S., Takahashi, S., Takebe, H., Takeshita, K., Tamasaku, K., Tanaka, H., Tanaka, R., Tanaka, T., Togashi, T., Togawa, K., Tokuhisa, A., Tomizawa, H., Tono, K., Wu, S., Yabashi, M., Yamaga, M., Yamashita, A., Yanagida, K., Zhang, C., Shintake, T., Kitamura, H. & Kumagai, N. (2012). *Nat. Photon.* **6**, 540–544.

[bb13] Kita, T., Harada, T., Nakano, N. & Kuroda, H. (1983). *Appl. Opt.* **22**, 512. 10.1364/ao.22.000512 18195819

[bb14] Li, Z. & Li, B. (2018). *J. Synchrotron Rad.* **25**, 738–747. 10.1107/S160057751800468X PMC5929356 29714183

[bb15] Li, Z. & Li, B. (2019). *J. Synchrotron Rad.* **26**, 1058–1068. 10.1107/S1600577519004648 PMC6613118 31274428

[bb16] Louis, E., Yakshin, A. E., Tsarfati, T. & Bijkerk, F. (2011). *Prog. Surf. Sci.* **86**, 255–294.

[bb100] Mankowsky, R., Subedi, A., Först, M., Mariager, S. O., Chollet, M., Lemke, H. T., Robinson, J. S., Glownia, J. M., Minitti, M. P., Frano, A., Fechner, M., Spaldin, N. A., Loew, T., Keimer, B., Georges, A. & Cavalleri, A. (2014). *Nature*, **516**, 71–73. 10.1038/nature13875 25471882

[bb17] McNeil, B. W. J., Thompson, N. R. & Dunning, D. J. (2013). *Phys. Rev. Lett.* **110**, 134802. 10.1103/PhysRevLett.110.134802 23581327

[bb18] Nicolas, J. & Cocco, D. (2022). *Photonics*, **9**, 367.

[bb19] Osborn, K. D. & Callcott, T. A. (1995). *Rev. Sci. Instrum.* **66**, 3131–3136.

[bb20] Penco, G., Allaria, E., Badano, L., Cinquegrana, P., Craievich, P., Danailov, M., Demidovich, A., Ivanov, R., Lutman, A., Rumiz, L., Sigalotti, P., Spezzani, C., Trovò, M. & Veronese, M. (2013). *J. Instrum.* **8**, P05015.

[bb21] Ragozin, E. N., Vishnyakov, E. A., Kolesnikov, A. O., Pirozhkov, A. S. & Shatokhin, A. N. (2021). *Phys.-Usp.* **64**, 495–514.

[bb22] Ratner, D., Abela, R., Amann, J., Behrens, C., Bohler, D., Bouchard, G., Bostedt, C., Boyes, M., Chow, K., Cocco, D., Decker, F. J., Ding, Y., Eckman, C., Emma, P., Fairley, D., Feng, Y., Field, C., Flechsig, U., Gassner, G., Hastings, J., Heimann, P., Huang, Z., Kelez, N., Krzywinski, J., Loos, H., Lutman, A., Marinelli, A., Marcus, G., Maxwell, T., Montanez, P., Moeller, S., Morton, D., Nuhn, H. D., Rodes, N., Schlotter, W., Serkez, S., Stevens, T., Turner, J., Walz, D., Welch, J. & Wu, J. (2015). *Phys. Rev. Lett.* **114**, 054801. 10.1103/PhysRevLett.114.054801 25699448

[bb23] Reiche, S., Musumeci, P., Pellegrini, C. & Rosenzweig, J. B. (2008). *Nucl. Instrum. Methods Phys. Res. A*, **593**, 45–48.

[bb24] Rife, J. C., Hunter, W. R., Barbee, T. W. & Cruddace, R. G. (1989). *Appl. Opt.* **28**, 2984. 10.1364/AO.28.002984 20555637

[bb25] Saldin, E. L., Schneidmiller, E. A. & Yurkov, M. V. (1998). *Nucl. Instrum. Methods Phys. Res. A*, **407**, 291–295.

[bb987] Sanchez del Rio, M., Canestrari, N., Jiang, F. & Cerrina, F. (2011). *J. Synchrotron Rad.* **18**, 708–716. 10.1107/S0909049511026306 PMC3267628 21862849

[bb103] Suga, M., Akita, F., Sugahara, M., Kubo, M., Nakajima, Y., Nakane, T., Yamashita, K., Umena, Y., Nakabayashi, M., Yamane, T., Nakano, T., Suzuki, M., Masuda, T., Inoue, S., Kimura, T., Nomura, T., Yonekura, S., Yu, L.-J., Sakamoto, T., Motomura, T., Chen, J.-H., Kato, Y., Noguchi, T., Tono, K., Joti, Y., Kameshima, T., Hatsui, T., Nango, E., Tanaka, R., Naitow, H., Matsuura, Y., Yamashita, A., Yamamoto, M., Nureki, O., Yabashi, M., Ishikawa, T., Iwata, S. & Shen, J.-R. (2017). *Nature*, **543**, 131–135. 10.1038/nature21400 28219079

[bb102] Takaba, K., Maki-Yonekura, S., Inoue, I., Tono, K., Hamaguchi, T., Kawakami, K., Naitow, H., Ishikawa, T., Yabashi, M. & Yonekura, K. (2023). *Nat. Chem.* **15**, 491–497. 10.1038/s41557-023-01162-9 PMC10719108 36941396

[bb26] Trzhaskovskaya, M. B., Nefedov, V. I. & Yarzhemsky, V. G. (2001). *At. Data Nucl. Data Tables*, **77**, 97–159.

[bb27] Voronov, D. L., Anderson, E. H., Cambie, R., Cabrini, S., Dhuey, S. D., Goray, L. I., Gullikson, E. M., Salmassi, F., Warwick, T., Yashchuk, V. V. & Padmore, H. A. (2011). *Opt. Express*, **19**, 6320. 10.1364/OE.19.006320 21451658

[bb28] Voronov, D. L., Goray, L. I., Warwick, T., Yashchuk, V. V. & Padmore, H. A. (2015). *Opt. Express*, **23**, 4771. 10.1364/OE.23.004771 25836513

[bb29] Wang, Y. & Li, B. (2021). *Nucl. Tech.* **44**, 080502.

[bb101] Wernet, Ph., Kunnus, K., Josefsson, I., Rajkovic, I., Quevedo, W., Beye, M., Schreck, S., Grübel, S., Scholz, M., Nordlund, D., Zhang, W., Hartsock, R. W., Schlotter, W. F., Turner, J. J., Kennedy, B., Hennies, F., de Groot, F. M. F., Gaffney, K. J., Techert, S., Odelius, M. & Föhlisch, A. (2015). *Nature*, **520**, 78–81. 10.1038/nature14296 25832405

[bb30] Wieland, M., Kabachnik, N. M., Drescher, M., Deng, Y., Arbelo, Y., Stojanovic, N., Steffen, B., Roensch-Schulenburg, J., Ischebeck, R., Malyzhenkov, A., Prat, E. & Juranić, P. (2021). *Opt. Express*, **29**, 32739. 10.1364/OE.432761 34809098

[bb31] Zhang, X., Guo, Z., Meng, X., Chen, J., Ji, Z., Jin, Z., Zhang, X., Wang, Y. & Tai, R. (2020). *J. Synchrotron Rad.* **27**, 870–882. 10.1107/S1600577520006554 33565995

[bb32] Zhao, Z., Wang, D., Gu, Q., Yin, L., Gu, M., Leng, Y. & Liu, B. (2017). *Appl. Sci.* **7**, 607.

